# Adopting a toolkit to manage time, resources, and expectations in the systematic review process: a case report

**DOI:** 10.5195/jmla.2021.1221

**Published:** 2021-10-01

**Authors:** Q. Eileen Wafford, Linda C. O'Dwyer

**Affiliations:** 1 q-wafford@northwestern.edu, Research Librarian, Galter Health Sciences Library and Learning Center, Feinberg School of Medicine Northwestern, Chicago, IL; 2 l-odwyer@northwestern.edu, Head of Research and Information Services, Galter Health Sciences Library and Learning Center, Feinberg School of Medicine Northwestern University, Chicago, IL

**Keywords:** systematic review, toolkit, expectations, team management, process management, project management

## Abstract

**Background:**

The proliferation of systematic reviews has impacted library operations and activities as librarians support, collaborate, and perform more tasks in the systematic review process. This case report describes a toolkit that librarians with extensive experience in supporting multiple review teams use to manage time, resources, and expectations in the systematic review process.

**Case Presentation:**

The toolkit is a compilation of documents that we use to effectively communicate with and help review teams understand and navigate each stage of the systematic review process. Elements included in the toolkit and discussed in this case report are intake forms, communication templates and memoranda, a process flow diagram, library guides on tools for retrieval and data appraisal, and established standards for guidance during the write-up stage. We describe the use of the toolkit for both education and project management, with a focus on its use in helping manage team time, resources, and expectations.

**Discussion:**

The systematic review toolkit helps librarians connect systematic review steps and tasks to actionable items. The content facilitates and supports discussion and learning by both librarians and team members. This toolkit helps librarians share important information and resources for each stage of the process.

## BACKGROUND

In 2000, 279 citations in PubMed had “systematic review” in their titles. That number jumped to over 22,300 in 2019. The proliferation of systematic reviews has substantially impacted library operations and activities as librarians support, collaborate, and perform more tasks in the systematic review process. This is evident in librarians' increasing involvement in systematic review projects as experts recognize that librarian involvement helps produce high-quality reviews [1]. In their 2003 study, Beverly et al. reported ten roles that librarians may perform as members of review teams [2]. In 2018, Spencer and Eldredge identified eighteen unique roles [3]. Galter Health Sciences Library and Learning Center offers two support models for systematic review teams: the consultant model and the full collaboration model. Teams that accept support under the consultant model meet with a librarian for a one-hour consultation where we discuss the process, tools, and provide tips on developing a comprehensive search. Review teams run their own searches and perform all tasks related to their review with minimal input from the librarian.

Galter librarians who assist teams under the full collaboration model perform tasks that include assisting teams with formulating a research question; searching for possible existing systematic or scoping reviews, including protocols, on the topic; identifying information sources and developing sensitive search strategies for each source; deduplicating search results and delivering them to the screening platform; assisting with full-text retrieval; documenting the search process; and writing the search methods for the protocol and manuscript. Librarians who partner with review teams as full collaborators commonly satisfy the criteria for authorship set forth by the International Committee of Medical Journal Editors [4]. Coauthorship is expected when a librarian serves as collaborator rather than a consultant.

Taking on the roles of instructor, methods expert, expert searcher, reference manager, document supplier, data manager, and author for each full collaborative review contributes to the time and resource-intensive requirements of systematic reviews. Bullers et al. found systematic review teams can invest up to 219 hours on a review [5]. The authors surveyed librarians and found they averaged around four hours for the initial consultations, over five hours on search strategy development and implementation, three hours on documentation, and two hours on writing [5]. This reflects the reality at Galter, as a research librarian often spends full days working on systematic review–related tasks. As we take on multiple roles and perform many tasks, often simultaneously and for multiple reviews, time, resources, and team management become significant challenges. Thus, in this case report, we describe a toolkit Galter librarians use to manage time, resources, and expectations in the systematic review process [6].

## CASE PRESENTATION

### The toolkit

The systematic review toolkit is a compilation of documents employed by Galter librarians to help teams understand and navigate the process of completing a systematic review. This toolkit contains the following:

- Intake form
- Memorandum of understanding (MOU)
- Email templates
- Systematic review process flow diagram [7]
- Preferred Reporting Items for Systematic Review and Meta-Analysis Protocols (PRISMA-P) checklist [8]
- PRISMA-P elaboration and explanation [9]
- Search strategy development document
- PRISMA 2009 statement [10]
- PRISMA explanation and elaboration [11]
- Examples of published protocols

We developed the toolkit by bringing together resources we use regularly in our systematic review support workflows and refined it through discussion at the library's Systematic Review Working Group (SRWG) meetings. All librarians who support systematic reviews attend monthly SRWG meetings where we share information, training, and experiences to promote best practices and standards. While some toolkit documents, such as the MOU and intake form, are required, we recognize review teams vary and not all need each document in the toolkit. Librarians at Galter have access to these resources and can use and adapt the toolkit's other resources as appropriate. The toolkit is intended to help us lead effective and efficient review teams at each stage of the process.

### Preconsultation

Each review project starts with knowledge gaps for each member of the team. The requesters know they want to conduct a review on a topic of interest and may have a vague idea of some of the mile markers to reach on the way to completing that review. As librarians, we are initially unaware of the topic's potential for a review and the reviewers' understanding of systematic review process. To close these early knowledge gaps, we start by providing and requesting information using a preconsultation email template, available in the toolkit (Appendix A). This email points to the library's systematic review guide, which contains links to core articles about the process and information on the different support models offered at Galter, both consultative and collaborative [12].

We provide an intake form for review teams to complete before the initial consultation (Appendix B). This form asks for information on the research question, members' experience with systematic reviews, relevant keywords, and any benchmark articles on their topic. There is an educational component as the intake form exposes reviewers to key elements of the systematic review workflow such as the patient, intervention, comparison, outcome (PICO) model; keywords/search strategy development; and articles that might help us validate the search. We use information from the intake form to understand the proposed review and prepare for the initial meeting by running preliminary searches for existing reviews. This baseline data—PICO, research question, search terms, potential filters or limits—form the basis for an initial consultation.

### Initial consultation

The initial consultation sets the tone for future communication and is the first real opportunity for librarians and potential collaborators to gain an understanding of each other's reservoir of knowledge. The foundation for the review's success, this meeting clarifies details such as the research question, protocol development, targeted databases, and timelines. Even with training materials and educational opportunities provided before the initial meeting, many reviewers approach this first consultation with limited knowledge of systematic reviews and lack understanding of the time needed to complete the review. Some members may have expectations based on their experience in previous systematic review projects, possibly at a different institution. To best illustrate the steps in a review, we use another resource from our toolkit: the systematic review flow diagram by Tsafnat et al. (Appendix C) [7]. This diagram communicates the complexity of conducting a systematic review in an easy-to-understand manner, which teams appreciate. Tsafnat et al.'s diagram identifies five phases or classifications of the systematic review process. These phases (Table 1), which may overlap, are preparation, retrieval, appraisal, synthesis, and write-up.

**Table 1:** Systematic review tasks, phases, and corresponding toolkit items

Task	Classification/phase	Toolkit item
1. Formulate review question	Preparation	Intake form
2. Find previous systematic review(s)	Preparation	Intake form, guide
3. Write the protocol	Preparation/write-up	PRISMA-P Checklist, PRISMA Elaboration and Explanation, protocol examples
4. Devise search strategy	Preparation	Intake form, search strategy document
5. Search	Retrieval	Intake form, guide
6. Deduplicate	Retrieval	SR process flow diagram, guide
7. Screen abstract	Appraisal	Intake form, email template, guide
8. Obtain full text	Retrieval	SR process flow diagram
9. Screen full text	Appraisal	Intake form, email template, guide
10. Snowball	Retrieval	SR process flow diagram
11. Extract data	Synthesis	Guide
12. Synthesize data	Synthesis	Guide
13. Re-check literature	Retrieval	SR process flow diagram
14. Meta-analyze	Synthesis	Guide
15. Write up review	Write-up	PRISMA 2009 Checklist, PRISMA flow diagram

Tsafnat et al. also highlight fifteen tasks that correspond to different phases of the process. Some librarians at Galter structure the initial consultation and map toolkit resources to elements in the diagram as illustrated in Table 1. Where appropriate, we note related tasks from Tsafnat et al.'s diagram to corresponding toolkit items.

### Preparation

Our preparation for a systematic review starts with the research question (Task 1). The most common problem we encounter at the outset of a systematic review is an overly broad research question. Sometimes there is no question, merely a topic that could yield more specific questions. Other times the question itself is too narrow or easily answerable with a quick search. We analyze the research question presented in the form in more depth and gauge its suitability for a systematic review. We may have already found an existing review (Task 2) addressing the proposed research question and can discuss options for moving forward. Our companion article on communication includes points for discussion around revising the research question or pursuing a question covered by an existing review [13].

We have observed that editors and peer reviewers are becoming increasingly familiar with the PRISMA checklist and will inquire about the protocol registration if it is missing from the manuscript as recommended by the PRISMA 2009 statement [11]. Our experience is that reviewers with published protocols are more likely to successfully complete and publish their systematic review. Consequently, protocol development (Task 3) and registration is required for teams interested in working with a Galter librarian under the full collaboration model. We send the PRISMA-P checklist and PRISMA elaboration and explanation document to help with protocol development.

The search strategy (Task 4) we develop is the cornerstone of the systematic review. If the intake form contains sufficient information to construct a preliminary search strategy, we present that search strategy document during the initial meeting. This helps them understand the development of a comprehensive search strategy and the role of keywords, subject headings, and possibly truncation, Boolean, and database proximity operators. By demonstrating the importance, details, and potential complexity of the search, we hope to convey that strategy development is an iterative process, requiring feedback from the reviewers. We will have questions during this phase, and the quicker reviewers respond to emails, the smoother the process and the better the search.

### Retrieval and appraisal

The transition from search strategy development to performing the searches (Task 5) is part of a larger conversation about information sources. Adapting and performing the search strategy to multiple databases is one of the most time-intensive tasks in the process [5]. Many reviewers are unfamiliar with the need to search multiple sources, the range of available sources, and the importance of grey literature [14]. The questions about information sources, including a list of major databases, on the intake form are one way to expose teams to the breadth of searching for a systematic review. Our systematic review guide also includes information about commonly searched databases, specialized databases, and grey literature sources.

Teams new to systematic reviews often have a limited understanding of the methods for screening records (Tasks 7 and 9) for a systematic review. Each member should leave the initial meeting with awareness of available screening tools like Covidence or Rayyan. At Galter, reviewers are responsible for setting up their reviews in these tools. We will use EndNote to compile results into a master library and will obtain full-text, available through our institutional subscriptions, open access, or for free. They submit any interlibrary loan requests required; we can facilitate that process by providing instructions on the most efficient way to obtain resources through document delivery.

### Synthesis and write-up

Some reviewers we work with are unfamiliar with data extraction (Task 11) and synthesis (Task 12), including risk of bias assessments and grading evidence. While we do not advise on choice of tools, our systematic review guide has various risk of bias checklists, quality assessment tools, and a template of a data extraction form for consideration. We similarly do not provide direct assistance with statistical support for the meta-analysis (Task 14); however, the guide provides links to units on campus and items from the library's catalog that can help with this aspect as needed.

In addition to the PRISMA-P checklist, the toolkit includes the PRISMA 2009 checklist, the PRISMA explanation and elaboration document, and an editable PRISMA flow diagram to support the manuscript write-up (Task 15). We recommend that reviewers use the PRISMA 2009 checklist as they write and edit their manuscript. At Galter, we appraise a draft of the manuscript using the PRISMA 2009 checklist to identify potential methodological issues and send reviewers that checklist with their edited manuscript. We usually provide a PRISMA flow diagram with a record of the search results and a search strategies appendix to be included as supplementary materials with the manuscript submission.

### Putting it all together

Once reviewers understand the process and resources involved in completing a systematic review, it is easier for us to talk about timelines. We find reviewers are more understanding of the time it takes to complete a systematic review after learning about the process using Tsafnat et al.'s diagram as a reference point. Moreover, reviewers are better able to understand their responsibilities and the librarian's responsibilities after review of the MOU, also found in the toolkit (Appendix D).

The amount of information covered during the initial consult can be overwhelming for team members. Reviewers feel reassured when they receive a follow-up email from us with attachments and links to the resources mentioned during these initial conversations. The postconsultation email template details key discussion points, links or attachments to relevant resources, and action items for the reviewers and for us (Appendix E). We also understand that we may need to describe these steps again over the course of the months-long, sometimes years-long, project—especially for individuals who are newer to the process. These early meetings are about clarification and exposure to the process.

## DISCUSSION

The launch of a systematic review is multifaceted with a constant flow of information and resources between us and the reviewers. Preconsultation materials give us a glimpse of the team's dynamic and knowledge of the systematic review process and their topic as well as the research question and its potential to produce a systematic review. In the initial consultation or consultations, we present and request information to train reviewers on the process, refine their research question, define our roles and responsibilities, and get them started on their review. Ensuring we miss nothing in the limited time allotted for a consultation can feel daunting. The systematic review toolkit helps us connect the process and tasks to actionable items supported by various resources.

Systematic review processes, services, and support may differ from institution to institution. For librarians interested in adopting the toolkit, we recognize the need to do so in a way that reflects the library and librarian's situation. Some toolkits might incorporate additional documents [15]. Furthermore, the order in which a librarian discusses each task might vary. The toolkit is still in a pilot phase at Galter. Each librarian at our library uses their own version of it, selecting elements and tailoring them to each project. Some librarians at Galter present the PRISMA-P checklist at the initial meeting, while other librarians provide these documents after several meetings. As we discuss our individual challenges and processes during departmental and SRWG meetings, we are developing a consensus on documents and tools that will make up a standardized, library-wide toolkit.

The processes used by Galter librarians are constantly evolving as we gain experience working with different systematic review teams, pursue training to become better searchers, and explore topics and issues related to systematic reviews in the library's SRWG. This results in a systematic review toolkit reflecting growth and progress in the systematic review process.

## Data Availability

There are no data associated with this article.

## SUPPLEMENTAL FILES

**Appendix A:** Email template (preconsultation)

**Appendix B:** Systematic review intake form

**Appendix C:** Steps in the systematic review process

**Appendix D:** Search strategy development template

**Appendix E:** Memorandum of understanding (MOU) for collaborative projects with Galter Health Sciences Library & Learning Center

**Appendix F:** Postconsultation(s) email template
