# Supplementary material for: Adopting a toolkit to manage time, resources, and expectations in the systematic review process: a case report
Source: J Med Libr Assoc. 2021 Oct 1;109(4):637–42. doi: 10.5195/jmla.2021.1221 (PMC8608198; doi:10.5195/jmla.2021.1221)
Supplement: Supplementary file 1 — Appendix A: Email template (preconsultation) [file jmla-109-4-637-s01.docx]

**APPENDIX A**

**Email template (pre-consultation):**

Hi NAME,

Thanks for getting in touch. I’d be happy to meet with you to answer your questions and talk about our systematic review services.

We have two support models [hyperlink to guide with a list of task performed under each model]: the consult model is where we meet with you once and talk about process, search terms, and recommended databases. The second option is a full collaboration where we meet, talk process, and help you focus your question. The librarian will draw up search terms, run the searches in each database, assist with finding PDFs, write the search methods for the protocol and manuscript, and assist in other related tasks. This support model takes more time and the librarian is listed as a co-author on the review.

If you’re interested in the full collaboration model, can you complete the form at [hyperlink to intake form]? This form is helpful in framing a focused research question and identifying concepts which can later be elaborated upon in the protocol and manuscript. Don’t worry if you have to leave some questions unanswered, we’ll go over the form in a future meeting.

Upon receiving the completed form, we can set up a meeting to discuss the review in more detail.

In the meantime, you can learn more about [preparing for your systematic review](https://galter.northwestern.edu/galterguides?url=https%3A%2F%2Flibguides.galter.northwestern.edu%2Fc.php%3Fg%3D517817%26p%3D3540652) and the [systematic review services at Galter](https://galter.northwestern.edu/galterguides?url=https%3A%2F%2Flibguides.galter.northwestern.edu%2Fsystematic-reviews%2Fservices) [here](https://galter.northwestern.edu/galterguides?url=https%3A%2F%2Flibguides.galter.northwestern.edu%2Fc.php%3Fg%3D517817%26p%3D3540634).

Looking forward to hearing from you,

Best,

LIBRARIAN NAME
