# Supplementary material for: Adopting a toolkit to manage time, resources, and expectations in the systematic review process: a case report
Source: J Med Libr Assoc. 2021 Oct 1;109(4):637–42. doi: 10.5195/jmla.2021.1221 (PMC8608198; doi:10.5195/jmla.2021.1221)
Supplement: Supplementary file 2 — Appendix B: Systematic review intake form [file jmla-109-4-637-s02.docx]

**APPENDIX B**

**Systematic Review Intake Form**

Thank you for considering Galter Library to assist with your review. This form will help us prepare for a consultation with you. If you do not have an answer, skip that question. A librarian will go over this form with you. To discuss your [review options](https://galter.northwestern.edu/galterguides?url=https%3A%2F%2Flibguides.galter.northwestern.edu%2Fsystematic-reviews%2Fservices) before completing this form, contact [Linda O’Dwyer](mailto:l-odwyer@northwestern.edu), Head, Research & Information Services or your [liaison librarian](https://galter.northwestern.edu/Research%20Services/liaison).

1. Name:
2. Email:
3. Phone:
4. NU Affiliation:
5. Department, Center, or Program:
6. Affiliation:
7. Are you the principal/first author?
8. Names of other colleagues on your team who are currently or will be working on the project:
9. Have you or your team completed other systematic or similar reviews?
10. What is the overall timeline for your project, i.e. start, finish, and additional milestone dates?
11. What type of review are you planning? (systematic, meta-analysis, scoping, other, unsure)
12. Have you consulted or worked with a librarian at Northwestern University or its affiliated hospitals on this project?

If yes, please provide the name of the librarian:

1. What is your research question?

If appropriate, please define your question using PICO (Problem, Intervention, Comparison, and Outcome) or PCC (Population, Concept, Context):

- Patient/Population/Problem:
- Intervention/Exposure/Interest/Concept:
- Comparison(optional):
- Outcome/Context:

Write your PICO or PCC in question form:

1. List synonyms and related words for each PICO component that can be used for the search.

| **Patient/Population** | **Intervention/Exposure/**  **Concept** | **Comparators**  **(Optional)** | **Outcome(s)/Context** |
| --- | --- | --- | --- |
|  |  |  |  |
|  |  |  |  |
|  |  |  |  |
|  |  |  |  |
|  |  |  |  |

1. Types of studies (publication types) **to include** in the review:

| Meta-analysis | Cohort Study | Editorials/Opinions |
| --- | --- | --- |
| Systematic Review | Case Control Study | Qualitative research |
| Randomized Controlled Trial | Case Series/Report | Other _______________ |

1. List any limits that may pertain to your search—e.g. gender, age, etc.:
2. Indicate databases of interest:

MEDLINE/PubMed Cochrane Library EMBASE CINAHL

PsycINFO Web Of Science Scopus Other(s)

1. Would you like to include any of the following?

   Grey literature: [check-boxes; check all that apply]

Theses and dissertations  Clinical trials registries  Conference proceedings

Specialty journals (provide names):

Specialty databases or sources, e.g. professional society websites, industry websites, etc.,

1. Pre-work:

(a) Please list 5-7 benchmark articles that ***meet the inclusion criteria*** (PICO or PPC) for your review. These articles should appear in the final analysis.

(b) Please provide the citations to systematic reviews published on this topic in the last 5 years:

(c) Please provide a link or citation to protocols in [Prospero](https://www.crd.york.ac.uk/prospero/) or elsewhere:

1. Have you [developed a protocol](https://galter.northwestern.edu/galterguides?url=https%3A%2F%2Flibguides.galter.northwestern.edu%2Fc.php%3Fg%3D517817%26p%3D6892395) using the PRISMA guidelines?
2. Screening platform:
    Covidence  Rayyan  Other __________  Unsure/need advice
3. Have you selected a target journal for publication? Y/N
   If yes, list the targeted journal(s):
4. Librarians collaborating on systematic reviews commonly satisfy [the criteria for authorship set forth by ICMJE](http://www.icmje.org/recommendations/browse/roles-and-responsibilities/defining-the-role-of-authors-and-contributors.html). Co-authorship is expected when a librarian serves as collaborator rather than a consultant. See how the [roles](https://galter.northwestern.edu/galterguides?url=https%3A%2F%2Flibguides.galter.northwestern.edu%2Fsystematic-reviews%2Fservices) differ.

The Galter librarian will be (co-author, consultant only, I’d like to discuss):
