# Supplementary material for: Adopting a toolkit to manage time, resources, and expectations in the systematic review process: a case report
Source: J Med Libr Assoc. 2021 Oct 1;109(4):637–42. doi: 10.5195/jmla.2021.1221 (PMC8608198; doi:10.5195/jmla.2021.1221)
Supplement: Supplementary file 3 — Appendix C: Steps in the systematic review process [file jmla-109-4-637-s03.docx]

**APPENDIX C**

**[Steps in the Systematic review process]**


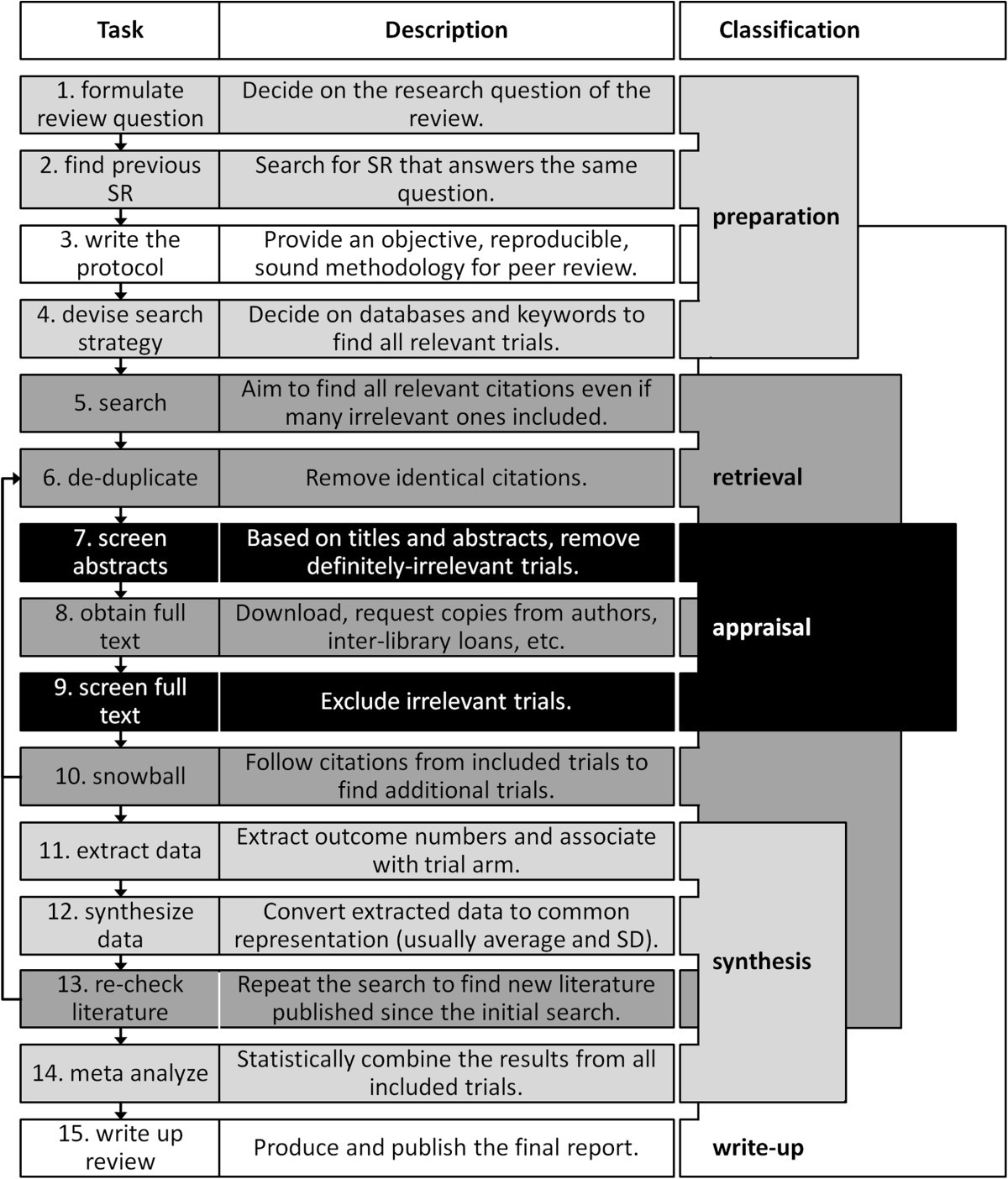


Source: Tsafnat, G., Glasziou, P., Choong, M.K. et al. Systematic review automation technologies. Syst Rev 3, 74 (2014). <https://doi.org/10.1186/2046-4053-3-74>
