# Supplementary material for: Adopting a toolkit to manage time, resources, and expectations in the systematic review process: a case report
Source: J Med Libr Assoc. 2021 Oct 1;109(4):637–42. doi: 10.5195/jmla.2021.1221 (PMC8608198; doi:10.5195/jmla.2021.1221)
Supplement: Supplementary file 4 — Appendix D: Search strategy development template [file jmla-109-4-637-s04.docx]

**APPENDIX D**

**SEARCH STRATEGY DEVELOPMENT TEMPLATE**

**Search Strategy Development**

Last updated – Date

For the systematic review search, we will combine Medical Subject Headings (MeSH) or its equivalent controlled vocabulary in other databases and keyword searches of titles and abstracts. This assures the broadest possible inclusion.

This is a working document with terms and concepts that will develop as we refine the research question, develop the protocol, and mine various information sources.

**Research question/topic:**

- E.g. What are methods used to measure the level of stress in health profession trainees (Doctors, nurses, paramedics,...)

**PICO**

- Patient/Population:
- Intervention/Exposure:
- Comparators:
- Outcome(s):

**Patient/Population:**

- MeSH
  - "Mesh Term"[Mesh]
- Keywords
  - Keyword 1
  - Keyword 2
- Questions/observations:
- PubMed Search String:

**Intervention/Exposure:**

- MeSH
  - "Mesh Term"[Mesh]
- Keywords
  - Keyword 1
  - Keyword 2
- Questions/observations:
- PubMed Search String:

**Comparator:**

- MeSH
  - "Mesh Term"[Mesh]
- Keywords
  - Keyword 1
  - Keyword 2
- Questions/observations:
- PubMed Search String:

**Outcome:**

- MeSH
  - "Mesh Term"[Mesh]
- Keywords
  - Keyword 1
  - Keyword 2
- Questions/observations:
- PubMed Search String:

**Combined PubMed Search String:**

**Reference Articles Not Found in Search:**

**Notes:**
