# Supplementary material for: Adopting a toolkit to manage time, resources, and expectations in the systematic review process: a case report
Source: J Med Libr Assoc. 2021 Oct 1;109(4):637–42. doi: 10.5195/jmla.2021.1221 (PMC8608198; doi:10.5195/jmla.2021.1221)
Supplement: Supplementary file 5 — Appendix E: Memorandum of understanding (MOU) for collaborative projects with Galter Health Sciences Library & Learning Center [file jmla-109-4-637-s05.docx]

**APPENDIX E**

**MEMORANDUM OF UNDERSTANDING (MOU) FOR COLLABORATIVE PROJECTS with Galter Health Sciences Library & Learning Center**

**Purpose and Scope**

This is an agreement between ___________________(“PARTNER”) and Galter Health Sciences Library & Learning Center __________________(“LIBRARIAN”) on ___________________("PROJECT TITLE").

Galter Library is committed to doing the best work possible on your project and we look forward to working with you. Collaborative projects are time and resource-intensive endeavors. The purpose of this MOU is to clearly identify the roles and responsibilities of each party as they relate to the project to ensure success for everyone.

**Definitions**

For the purposes of this MOU, a collaborative project is defined as any one of the following: a systematic, scoping, integrative, network, or umbrella review; a meta-analysis; a systematic search required for a grant, either proposed or awarded; a systematic search required to create practice guidelines. For other collaborative projects not mentioned, please consult your librarian to see if they fit this scope and if this MOU applies.

**Responsibilities**

1. **Partner**
2. Meets with the librarian before commencing the project to establish search parameters such as resources/databases, search terms, and other inclusion/exclusion criteria
3. Develops a project protocol and registers the protocol in PROSPERO or another recognized registry or journal (if a systematic review) or DigitalHub (if other review type)
4. Screens search results according to established standards
5. Completes analysis of included studies according to established standards
6. Includes the librarian in team meetings when relevant
7. Keeps the librarian involved in the project communication from beginning to completion
8. Makes interlibrary loan requests for full-text items the library is unable to access (additional charges may apply)
9. Sends the full manuscript (including figures, tables, and supplementary materials) to the collaborating librarian for review before journal submission
10. Includes the librarian as a co-author on all scholarly outputs
11. Informs librarian as soon as possible if the project is significantly delayed or terminates unexpectedly
12. **Librarian**
13. Meets with the research team to plan systematic search methods
14. Develops an appropriate search strategy based on the research question
15. Collaborates with the team in developing the protocol
16. Conducts the literature searches and provides search results in an agreed upon format
17. Assists in the organization of retrieved references, and management of reference and/or review software
18. Keeps the research team informed on progress of search activities and updates
19. Maintains records of search results and follow up with alerts and updates as needed during the project timeline
20. Performs automated full-text retrieval and provides PDFs to the project team
21. Provides training to project­­­ team's administrative staff or designated team member for manual full-text retrieval (i.e. accessing additional full text articles or utilizing the Galter Library interlibrary loan service)
22. Documents all work and assists with the writing process, particularly the search methods section, and adds other relevant documentation (e.g., search strategies, data for PRISMA flow diagram)
23. Informs the researcher of any delays in meeting deadlines or of other changes that may affect provision of these responsibilities
24. **Funding & Partnerships**

Will you apply for grant funds to support this project? Yes ______ No ________

We would be delighted to provide a letter of support to include with your application. Please send any relevant information and your timeline to help us prepare a strong letter.

Interested in learning how we can partner on your next grant application or ongoing project? Please contact [galter-admin@northwestern.edu](mailto:galter-admin@northwestern.edu) to discuss your needs.

Effective Date and Signatures

______________________________ __________________

Library representative and title Date

______________________________

Email

______________________________ __________________

Partner Name and title Date

______________________________

Email
