# Supplementary material for: Adopting a toolkit to manage time, resources, and expectations in the systematic review process: a case report
Source: J Med Libr Assoc. 2021 Oct 1;109(4):637–42. doi: 10.5195/jmla.2021.1221 (PMC8608198; doi:10.5195/jmla.2021.1221)
Supplement: Supplementary file 6 — Appendix F: Postconsultation(s) email template [file jmla-109-4-637-s06.docx]

**APPENDIX F**

**Post-consultation(s) email template**

Hi NAME,

It was great meeting with you yesterday and learning more about your research! I’ve attached the following documents for review:

- Steps in the systematic review process
- PRISMA-P checklist to create the protocol
  - See Galter’s [Protocol Development](https://galter.northwestern.edu/galterguides?url=https%3A%2F%2Flibguides.galter.northwestern.edu%2Fc.php%3Fg%3D517817%26p%3D6892395) guide for more tools
- PRISMA-P 2015:elaboration and explanation
- PRISMA 2009 checklist for writing up your systematic review
- Memorandum of Understanding

To recap our conversation, we will generate a list of candidate terms consisting of keywords/“natural language” words and MeSH terms for the following concepts:

- Concept A –
- Concept B –
- Concept C –
- Concept D –

Please note these concepts may change based on information provided in the protocol.

We will search the following databases:

- MEDLINE (Ovid)
- Cochrane Library (Wiley)
- Scopus (Elsevier)
- CINAHL Plus with Full Text (Ebsco)
- PsycInfo (Ebsco)
- Clinicaltrials.gov (results sent as a URL)

Online resources covered in the meeting:

- Galter’s [Reporting Research and Evaluating Studies](https://galter.northwestern.edu/galterguides?url=https%3A%2F%2Flibguides.galter.northwestern.edu%2Fc.php%3Fg%3D853725) guide for links to risk of bias checklists
- Rayyan -  <https://rayyan.qcri.org/welcome> (screening tool)
- Covidence - <https://www.covidence.org/> (screening tool)
- PROSPERO - <https://www.crd.york.ac.uk/prospero/> (where you will register your protocol)

Action Items:

- Team: Sign and return the MOU
- Team: Draft the protocol (I will provide information for items 9 and 10)
- Librarian: Look for existing systematic reviews in Cochrane, Prospero, Medline
- Librarian: Compile a list of MeSH terms and keywords for each concept (to be finalized upon reviewing the protocol)
- Librarian: Draft a write up of the search strategy for the protocol

Please let me know if you have any questions.

Thanks!

LIBRARIAN NAME
